# Supplementary material for: Structural maintenance of chromosomes (SMC) proteins are required for DNA elimination in Paramecium
Source: Life Sci Alliance. 2023 Dec 6;7(2):e202302281. doi: 10.26508/lsa.202302281 (PMC10700549; doi:10.26508/lsa.202302281)
Supplement: Supplementary file 1 [file LSA-2023-02281_TableS1.docx]

| Oligo name | Sequence 5'-3' |
| --- | --- |
| SMC4-1 +f R | TCAACATGAATAATTAATTTTAAATTCTTCATTCTT |
| SMC4-1 +f F | AAAGAATTAACTAGTATTTTGCAGAAAAAATATTAAG |
| SMC4-1 +fv 3 F | TGAAATGATTTTAAAATATTCATATTCCATTATTG |
| SMC4-1 +fv 3 R | TCTATTTTAATTAACGTTTTCATTGTTTTC |
| SMC4-1 +fv F | CAGACTGAAAGTGAAGAGCCAT |
| SMC4-1 +fv R | CATTTTATGTAAATATATTTAATATTTTTAAATTAACTTTTGG |
| SMC4-2 +F R | GAATTAATTCAAGATCTAAATATTCGTAACA |
| SMC4-2 +F F | ATGCGCAATTTAATTTGAGCAATATT |
| SMC4-2 +fv 3 F | TGAAATTTATATTAAATTATAATCAATTAATTTTATC |
| SMC4-2 +fv 3 R | AACTTCTAATTAGATGACTTCTGTTGAA |
| SMC4-2 +fv F | ATTAAGGAAGTTATTTTGGAGAATTTTAAATC |
| SMC4-2 +fv R | CATATTTTCTATCATTTAATTTTCTACTCAAA |
| SMC2-1 sil F | ATATGAGCTCATGTGGATCAAAGAAATCATTATCGA |
| SMC2-1 sil R | TACCAGGAGACAAAAAGAAACTATAGACTCGAGTAAA |
| SMC2-2 sil F | ATATGAGCTCAGAACAACTGAATAGAGAGATTACACA |
| SMC2-2 sil R | AAAAGAATTCTAATCATTAAAAGACAAAAAGTTGCTCGAGTAAA |
| SMC4-1 sil F | ATATGAGCTCATGCAGACTGAAAGTGAAGAGC |
| SMC4-1 sil R | TTTACTCGAGTGTTTTGTTATGATGTCAGATTGTTCACT |
| SMC4-2 sil F | ATATGAGCTCATGATTAAGGAAGTTATTTTGGAGAATTTT |
| SMC4-2 sil R | TTTACTCGAGTATCTACCTTATTTTATTTTTCTGGAATCA |
| SMC2-1 Co-sil F | ATATCCCGGGATGTGGATCAAAGAAATCATTATCGA |
| SMC2-2 Co-sil F | ATATCCCGGGAGAACAACTGAATAGAGAGATTACACA |
| SMC4-1 Co-sil F | ATATCCCGGGATGCAGACTGAAAGTGAAGAGC |
| SMC4-2 Co-sil-R | TTTACCCGGGTATCTACCTTATTTTATTTTTCTGGAATCA |

**Supplementary Table S1.** Oligo sequences used for the study.
